# Effects of Climate Change on Ticks and Tick-Borne Diseases in Europe

**DOI:** 10.1155/2009/593232

**Published:** 2009-01-04

**Authors:** J. S. Gray, H. Dautel, A. Estrada-Peña, O. Kahl, E. Lindgren

**Affiliations:** ^1^School of Biology and Environmental Science, University College Dublin, Belfield, Dublin 4, Ireland; ^2^IS Insect Services GmbH, Haderslebener Straße 9, 12163 Berlin, Germany; ^3^Department of Parasitology, Veterinary Faculty, University of Zaragoza, Miguel Servet 177, 50013 Zaragoza, Spain; ^4^Applied Zoology/Animal Ecology, Institute of Biology, Free University of Berlin, 12163 Berlin, Germany; ^5^Stockholm Resilience Centre, Stockholm University, 106 91 Stockholm, Sweden

## Abstract

Zoonotic tick-borne diseases are an increasing health burden in Europe and there is speculation that this is partly due to climate change affecting vector biology and disease transmission. Data on the vector tick *Ixodes ricinus* suggest that an extension of its northern and altitude range has been accompanied by an increased prevalence of tick-borne encephalitis. Climate change may also be partly responsible for the change in distribution of *Dermacentor reticulatus*. Increased winter activity of *I. ricinus* is probably due to warmer winters and a retrospective study suggests that hotter summers will change the dynamics and pattern of seasonal activity, resulting in the bulk of the tick population becoming active in the latter part of the year. Climate suitability models predict that eight important tick species are likely to establish more northern permanent populations in a climate-warming scenario. However, the complex ecology and epidemiology of such tick-borne diseases as Lyme borreliosis and tick-borne encephalitis make it difficult to implicate climate change as the main cause of their increasing prevalence. Climate change models are required that take account of the dynamic biological processes involved in vector abundance and pathogen transmission in order to predict future tick-borne disease scenarios.

## 1. Introduction

Zoonotic tick-borne diseases in Europe have become increasingly prominent since the emergence of Lyme borreliosis (LB) in the early 1980s, and the incidence of this disease and that of tick-borne encephalitis (TBE) have risen dramatically over the last two decades [1]. Both diseases are transmitted by hard ticks of the *Ixodes ricinus* species complex (*I. ricinus* and *I. persulcatus)* and since these ticks spend most of their time in the environment, climate change is likely to affect their distribution and abundance and, therefore, the incidence of disease. *I. ricinus* and *I. persulcatus* are particularly sensitive to environmental conditions since in their prolonged nonparasitic phases they require a microclimatic relative humidity of at least 80% to avoid fatal desiccation. They are, therefore, restricted to areas of moderate-to-high rainfall where there is a good cover of vegetation, so that the soil surface remains humid through the driest times of the year [2]. The Fourth Assessment Report of the Intergovernmental Panel on Climate Change [3] reported that in northern temperate Europe temperature increases of 1.5–2.5°C may occur over the next few decades as a result of global warming. Such climate change may extend or curtail host-seeking tick activity periods, potentially increasing or decreasing tick abundance and distribution, and effects on tick development rates can change seasonal activity patterns by altering the proportion of the tick population that are exposed to regulatory mechanisms such as diapause. In areas where lowered summer precipitation coincides with raised summer temperatures, the survival, activity, and distribution of *I. ricinus* and *I.* *persulcatus* are likely to be reduced because of their vulnerability to desiccation. These tick species acquire their hosts by ambushing them from the vegetation and a significant number of large animals, such as deer, must be present in the habitat in order to feed the adult females and thus maintain the tick populations. The more catholic host preferences of the immature tick stages (larvae and nymphs), which parasitize reptiles, small and medium-sized mammals and birds, in addition to large mammals, contribute significantly to the circulation of diverse pathogens between the tick and host populations. Climate change may, therefore, exert a major influence on both tick abundance and disease prevalence by affecting faunal diversity [4].

Other important European tick species such as *Rhipicephalus sanguineus* and *Dermacentor reticulatus* can be affected by climate change through similar mechanisms, but anthropogenic factors also have profound effects on disease incidence, and separating these from the influence of climate change represents a major challenge. In this review attention is mainly focused on evidence for the effects of climate change on the distribution and abundance of European ticks. The potential impacts of climate-change effects on the incidences of the diseases they transmit are discussed.

## 2. Effects of Climate Change on Tick Distribution and Abundance

### 2.1. Ixodes ricinus

The climate is regarded as the principal restricting factor at the northern limit of *I. ricinus* distribution [5]. Although *I. ricinus* is surprisingly cold-hardy and when winter-acclimatized can survive 24-hour exposure to temperatures ranging from −14.4°C to −18.9°C [6], the detrimental effects of cold are accumulative and exposure for 30 days to only −10°C has been shown to be lethal for a high proportion of unfed nymphs and diapausing engorged larvae and nymphs [6]. Molting *I. ricinus* ticks are even more vulnerable so that if summer temperatures are not high enough to complete development before the onset of winter (little or no development takes place between 7 and 10°C [7, 8]), they are unlikely to survive even moderate frosts. Degree-day models have been developed which proved useful in elucidating the *I. ricinus* life cycle [9, 10], but more research on the winter biology of this species would help further understand its northern distribution limit.

**Figure 1:**
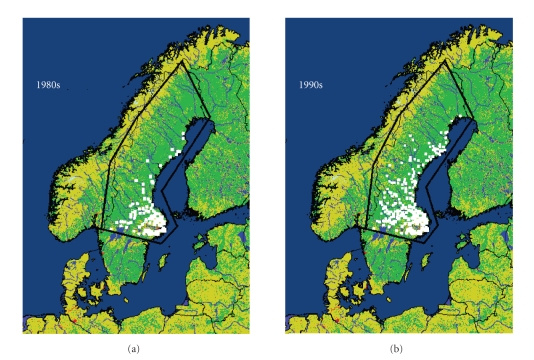
Changes in tick distribution in northern and central Sweden. White dots illustrate districts in Sweden where ticks were reported to be present before 1980 (a) and in 1994-1995 (b). The study region is within the black line (Lindgren et al. 2000, [12] with permission from *Environmental Health Perspectives*).

In Sweden, the northern distribution limit of *I. ricinus*, together with that of several other animal and plant species, has shifted northwards since the climate started to noticeably change in the late 1980s [11]. The geographical distribution range of *I. ricinus* used to be located below 61°N [5], but ticks are now established along the whole Baltic Sea coastline (up to 66°N) and along the river valleys and the larger lakes in the northern regions. This shift in latitude distribution has been shown to be related to changes over several seasons in the number of degree-days with temperatures vital for tick survival, activity and development [12]. At the highest latitudes fewer days with cold winter temperatures (well below −12°C for longer periods) had the clearest impact for new tick establishment (Figure 1).

In central Sweden (59°N to 61°N) in areas with medium-high densities of ticks, increases in tick abundance were correlated to a combination of mild winters (fewer days with temperatures below 7°C) and extended spring and autumn seasons (more days with minimum temperatures not lower than 5 to 8°C). In south and central Sweden, the current climate only allows a tick activity season of 6–8 months compared to as much as 11 months in some parts of the British Isles, and further changes in seasonal climate in Sweden are likely to continue to have a major impact on the prevalence of ticks. The combination of climate variables affecting ticks has also been shown to be significantly correlated with increases in the incidence of TBE in Stockholm County (59.2°N) during the period 1960–1998 [13]. The risks of LB are likely to increase as well because *Borrelia burgdorferi* sensu lato (s.l.) spirochetes have been found in *I. ricinus* throughout its range in Sweden [14].

A comparable situation has been reported in changes to the altitude distribution of *I. ricinus* in the mountainous regions of the Czech Republic. Field studies in 1957 and in 1979-80 showed that ticks were prevalent up to 700 meters above sea level. Ticks were collected from dogs or by drag-sampling in the same locations in 2001 and 2002 and were then found as high as 1100 meters in areas where they had been absent from small mammal samples [15–17] and where it has been shown that they could not complete their life cycle over the period 1957–1983 [18]. Furthermore, the prevalence of ticks carrying the TBE virus or *B. burgdorferi* s. l. spirochetes also seems to have increased at high altitude in the Czech Republic [16, 19].

### 2.2. Dermacentor reticulatus

*Dermacentor reticulatus* is a vector of canine babesiosis, tularemia, Q-fever, and at least one zoonotic rickettsiosis [20] and has vector competence for *Anaplasma marginale* [21]. Unlike *I. ricinus*, only the adults quest for hosts on surface vegetation and they feed primarily on deer, frequently bite dogs, but only occasionally humans, whereas the larvae and nymphs parasitize rodents. The life cycle is much shorter than that of *I. ricinus*, with eggs deposited in the spring and developing to adults within the same year [22]. The geographic range of the species extends from France and southwest England in the west to central Asia in the east. In western and central Europe its northern limit is northern Germany, northern Poland, and Lithuania, and its southern limit is the Mediterranean shore (restricted to humid mountainous areas), whereas it has a more northern distribution in the east (St. Petersburg). Within this area, its distribution is highly focal, and within Germany in 1976, it was only reported from four sites out of more than 3000 [23].

In two recent studies, however, data were collected showing that this tick species has since colonized many more sites in Germany [19]. The first study (2003) consisted of the screening of 365 dogs from 171 sites. Almost 10% of the ticks from 41 dogs were *D. reticulatus* and the infested dogs came from 26 sites, all previously unknown for the tick. Seven of the sites were subsequently confirmed by flagging. In the second study (2004), 721 deer were shot at 201 sites from 161 districts and their heads examined for ticks. A total of 23 (3.2%) deer from 14 sites were infested and only two sites were already known for *D. reticulatus.* These results strongly suggest that *D. reticulatus* has expanded its range within the last three decades particularly in the eastern and southwestern parts of Germany. Further evidence for the changing distribution of *D. reticulatus* is provided by the occurrence of canine babesiosis in new areas of Germany [24, 25], Hungary [26], Switzerland [27], and the Netherlands [28].

Several factors, perhaps acting synergistically, could be responsible for this recent spread of *D. reticulatus*, including increased deer abundance and the availability of more fallow land as a result of EU agricultural policies. However, there are good reasons for implicating a warming climate as being at least partly involved. Habitats with adult *D. reticulatus* are all characterized by more or less intense solar radiation and it is likely that the temperature sum (cumulative day-degrees above the developmental zero within one vegetation period) at the soil surface is a limiting factor for oviposition and embryonic development [20]. The fact that *D. reticulatus* occurs further north in eastern Europe supports this view, since the continental climate there is characterized by higher summer temperatures. Whereas adults are cold-hardy and can survive continental winters [29], eggs and larvae that do not complete development would not have survived the cold season of a few decades ago in central Europe [30]. Further research to clarify the situation could focus on suitable habitats in the north of Germany. 

### 2.3. Hyalomma marginatum

*H. marginatum* is well known as a vector of the dangerous viral zoonosis Crimean-Congo hemorrhagic fever (CCHF) [31]. Its possible northern spread and establishment of permanent populations is thus of much significance, especially since immature stages are frequently found on migratory birds flying to temperate Europe [32]. The life cycle of this tick is faster in southern parts of its distribution range (Northern Africa) [32] with larvae active as early as February, but clearly slower in northern, colder regions, with immatures active as late as June. Analysis of the recorded distribution of the tick show that, according to climate requirements, there are two clear clusters of populations [33]. One cluster extends from the northern geographical limit of the species in the Balkans (approx., latitude 44°N) and into Turkey and the Middle East. The second one is restricted to Africa north of the Sahara and western parts of Spain. Analysis of the climate niche of the first cluster clearly points to a temperature-related limiting factor for these northern populations. Temperatures between September and December are critical for the establishment of permanent populations. Cumulative temperatures between September and December have an average of 800°C in places where the tick has permanent populations, and below 400°C in sites not colonized by *H. marginatum*. This finding seems to be related to the factors that affect molting of immature stages and are not connected to the extremely cold winter temperatures that prevent overwintering adults surviving into the next year, as suggested by Hoogstraal et al. [32]. If temperatures are high enough to allow molting before the cold winters, unfed adults can survive the next active season. Field observations (Z. Vatansever, personal comment) recorded the feeding of nymphs in late summer in Turkey, with the resulting unfed flat adults commonly overwintering in the first few centimeters below the soil surface. Regulatory variables for these northern populations appear to act on thermodependent phases of the tick life cycle. On the other hand, climate niche analysis of the southern cluster of tick communities points to a strict dependence on rainfall and potential evaporation, but this may not be relevant if specimens from the southern range can adapt to the colder conditions of the northern cluster.

Although migratory birds are carriers of immature *Hyalomma* ticks and could potentially introduce them into currently *Hyalomma*-free areas in the spring, their climate requirements and current climate data do not suggest that they can become established. Mid-March and early April are the main periods of mass arrival of birds in Spain on their way to northern Europe. Data obtained from the Climate Research Unit (UK) show that average temperature in that period is 16-17°C in Morocco and Mauritania, 9–11°C in southern Spain, and 5-6°C in southern Germany. According to data on molting of engorged nymphs under laboratory conditions, about 300°C cumulative degrees above the developmental zero (14–16°C) are necessary to complete the molt [34]. Nymphs that engorge at the time of migratory bird arrival in early spring would need much longer to molt in southern Germany, with a consequent increase in mortality, than in north-western Africa, where only a few weeks are required. In the current climatic conditions, it is highly improbable that engorged nymphs can survive in sufficient numbers to be founders of new permanent populations in Europe. Immature *H. marginatum* are found on local (nonmigratory) birds in central Spain around late May and early June, which is too late for northern African and southern European populations of *H. marginatum* to mix because of the current climate barriers imposed by their respective climate requirements at the moment of bird migration. If climate change includes the predicted temperature increases, *H. marginatum* ticks may become established in northern latitudes but it is debatable whether initial introduction will occur as a result of bird migration alone because very small numbers of ticks, all immature stages, would be involved. It is more likely that, as autumn and winter temperatures rise, establishment of *H. marginatum* will mainly result from the introduction of adult females feeding on wild and domestic ruminants via the Middle East and the Balkans, where there is much uncontrolled movement of livestock.

### 2.4. Rhipicephalus sanguineus

A special case of distribution and association to environmental variables is that of the brown dog tick, *Rhipicephalus sanguineus*. It has a worldwide distribution mainly because of introduction by dogs, but rarely occurs in temperate and cold regions. However, *R. sanguineus* is an endophilous tick (associated with shelters like kennels, private gardens, or cracks in walls of human constructions), so may potentially cause temporary infestations in heated accommodation anywhere in the world. Within its normal range, *R. sanguineus* can reach huge populations under adequate environmental conditions and continued presence of a blood source. Currently, the brown dog tick is extremely common around the Mediterranean region. In the coldest places of this region, the tick may undergo a winter dormancy within the cracks of the walls, while in localities with warmer winters continuing activity may take place. Only sporadic cases of infestation by *R. sanguineus* have been described in central and northern Europe.

Studies on infestations in some Mediterranean cities [35] showed that permanent populations of the tick are absent in apartments where dogs are present, even without any kind of ixodicide treatment. However, the tick is present and may occur in large numbers in small private gardens and kennels of houses (or even within houses) in the outskirts. These country-type houses are very common in the Mediterranean region. Hourly climate data recorded by probes installed indoor and outdoor in these different types of construction showed that adequate humidity is a critical factor for successful establishment of indoor populations [35]. In central Europe, there are no humidity restrictions for the development of the tick in the private gardens or kennels and spring and summer temperatures are the only limitation. Recent studies on climate features [36] have shown that particular events, such as the heat wave in Europe in 2003, can result in temporary conditions adequate for the development and molting of immature stages. It is clear that despite the endophilous nature of this tick species, climatic conditions in the outer environment are critical for its long-term establishment in an area. An increase of about 2-3°C in the average temperature from April–September could result in the establishment of permanent populations of the tick in regions of northern temperate Europe where it is currently absent.

## 3. Effects of Climate Change on the Seasonal Aactivity of Ticks

### 3.1. Winter Activity of Ixodes ricinus

**Figure 2:**
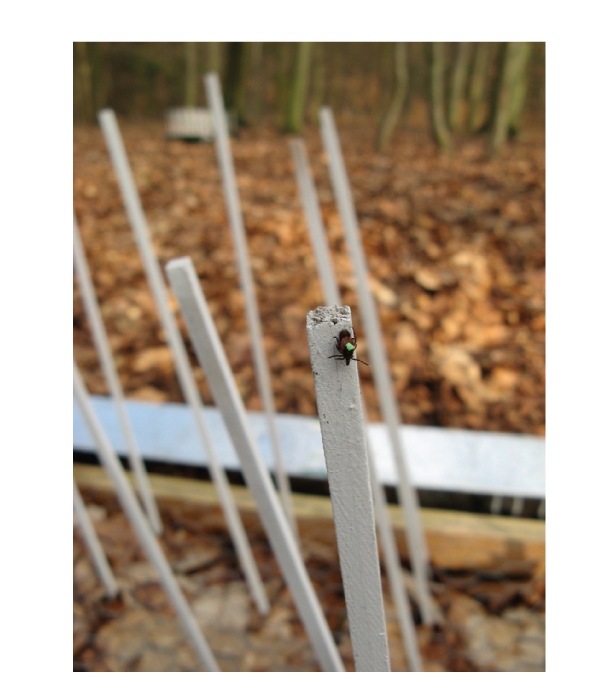
Dorsally marked adult female *Ixodes ricinus* questing on a wooden rod placed in a field plot for observation of *I. ricinus* questing activity in a Germany (Berlin) forest (Dautel et al. 2008 [37], with permission from *International Journal of Medical Microbiology*).

It is common knowledge that the seasonal activity of *I. ricinus* nymphs and adults extends from March to October in most parts of central Europe, whereas the larval stage begins questing only in May, at least in Berlin forests (Kahl and Dautel, unpublished). In contrast to parts of the British Isles, any tick activity from mid-November to mid-February is unusual in that region. The strong influence that temperature can exert on tick activity patterns, even in the cold season, was demonstrated in eastern Germany in the extraordinarily mild winter of 2006/7. The continuously warm-to-mild autumn in 2006 (1st September to 30th November), which was 3.4°C warmer than the long-term mean for 1961–90, was followed by a winter (1st December to 28th February) 4.6°C milder than the long-term mean (http://www.dwd.de, data from Potsdam) with only two days with a maximum temperature <0°C. On prepared field plots in a Berlin forest, questing adult *I. ricinus* (Figure 2) were found on every observation date throughout the winter (early November to early March) and questing nymphal ticks were absent on only two out of 19 occasions [36]. Moreover, Dautel and colleagues collected 88 nymphs and seven adult *I. ricinus* in two man-hours by flagging 1000 m^2^ in a nearby forest in mid-January 2007. At the same locality on another occasion in mid-February, nymphal and adult questing tick abundance was still higher (temperature maximum on both days approximately 7°C).

This is a good example of how flexible the seasonal questing activity of this widespread vector tick can be if the temperature conditions change from the local norm. The unusual registration of four cases of human TBE (a notifiable disease in Germany) in early 2007 (early January to late February) shows that there was some tick questing activity in other parts of Germany as well during that winter. If winter temperatures generally increase in the future, it can be assumed that seasonal periods with no questing *I. ricinus* will become shorter in central Europe. It is evident that winter activity of vector ticks distinctly increases the risk for forest visitors of an infectious tick bite, especially if they do not expect ticks to be active at that time of the year. It is unclear, however, what the chances are for a winter-active tick to find a host at that time (because of reduced host abundance in winter though this may also change with the advent of warmer winters). It is also unclear how winter activity may affect the remaining seasonal activity pattern (winter-active ticks spend precious energy) or whether any changes in the seasonal activity of *I. ricinus* nymphs and adults are beneficial or detrimental for the perpetuation of tick-borne pathogens. The infection of *I. ricinus* larvae during feeding is a crucial step in the circulation of many tick-borne pathogens and the effects of higher ambient temperatures on the seasonal activity of larvae and its chances to find a suitable host may be of great significance in determining the prevalence of some tick-borne diseases.

### 3.2. Summer Activity of Ixodes ricinus

In 1976 the early summer weather in County Wicklow, Ireland, included maximum air temperatures of 29–31°C recorded on sheep pastures where *Ixodes ricinus* activity was being studied. Such monthly maxima in early summer may be the rule rather than the exception in this region in the coming decades [3]. Retrospective use was made of 1976 summer temperature and tick data in an examination of the effects of high temperatures on tick development and activity in relation to the predicted global warming [38]. In most parts of its range, *I. ricinus* shows some degree of bimodal seasonal activity and in 1975, more ticks were collected in autumn than in spring/summer, which may be attributed to the presence of hosts on these particular sheep pastures in late summer and autumn but not in spring or early summer for several years. However, by 1977, the pattern of tick activity had changed dramatically and more than 90% nymphal activity occurred from March to June. It was postulated that the elevated early and mid-summer temperatures of 1976 were the primary cause of the change from autumn to spring/summer-dominated nymphal activity.

This possibility was investigated by studies on tick development under quasi-natural conditions. The threshold period for deposited engorged larvae to enter a developmental diapause was identified as the first two weeks of August, after which time larvae overwintered in an engorged state and did not reappear as nymphs until the following autumn. The tick abundance data suggested that the 1975 autumn-feeding adults gave rise to larvae that fed predominantly in the prediapause period, so that they had the opportunity to overwinter as unfed nymphs and thus join the spring-active ticks in 1977. This interpretation was supported by a degree-day development model for *I. ricinus* originally described by Gardiner et al. [9], that predicted the appearance of larvae from autumn-laid eggs a month earlier when exposed to 1976 temperatures than when exposed to more normal temperatures [10]. The high summer temperatures of 1976 had apparently transferred ticks from an autumn-active cohort to a spring-active one. The process revealed by this study suggests that after hotter summers much of the host-seeking activity of *I. ricinus* will occur in late autumn, to a lesser extent in the winter months, and with strong activity again in early spring. Larval activity is likely to be mainly restricted to mid-summer (as long as humidity requirements are satisfied) with the majority of larvae avoiding developmental diapause and becoming active as nymphs in late autumn or early spring of the following year. Interestingly, this pattern of activity is very similar to that of the American *I. scapularis* in New Jersey, USA [39, 40], where air temperatures exceed 26°C for 50–60 days of the year. Suitable studies in southern Europe have not been undertaken, but a similar phenology to the predicted scenario for *I. ricinus* in hot summers was described in a recent comprehensive study in central Spain where maximum summer air temperatures generally reach 26°C [41]. It might be expected that with low precipitation in hot summers, survival and activity of ticks such as *I. ricinus,* which is very susceptible to desiccation, would be reduced. Indeed, it has been reported that in Switzerland saturation deficits depressed nymphal and adult *I. ricinus* activity [42]. However, Irish data [38] showed that all active stages of *I. ricinus* will quest throughout hot dry weather as long as appropriate vegetation cover is present to provide opportunities for rehydration. The same situation appears to obtain for *I. scapularis* immature stages in the USA [40].

It seems likely that with increased global warming, *I. ricinus* activity will occur more in the autumn and winter months in many areas and, furthermore, a greater proportion of the tick population may be active at this time than at present, with a consequent temporal change in the risk of tick-borne diseases.

## 4. The Role of Modeling in Analysis of Climate Change Effects on Ticks

### 4.1. Climate Suitability Modeling

Climate suitability for a tick population can be defined as the fitness of a set of climatic conditions for the existence of that population in a given region. However, many other factors operating at different levels restrict the effective dispersal and establishment of potential invaders. Thus, while the climate in a particular location may be suitable for a given tick species, the potential for dispersal there and the ability to establish a new viable population may be very low. Furthermore, microclimatic variables such as soil surface temperature and relative humidity (which are affected by such things as slope and aspect, snow cover, vegetation, litter layer, humus and underlying soils) may be crucial in determining the distribution pattern of specific niches for tick survival within an area. Most data on climate preferences of ticks have been empirically derived from descriptions of the abiotic components of the environmental niche, as defined by the climate-supported native populations, and based on the assumption that they are homogeneously distributed in the native area.

The basic concept underlying species occurrence modeling is the definition of the ecological niche: each species is found within specific ranges for environmental variables that support individual survival and reproduction [43]. We refer here to climate instead of environmental or ecological space because these studies are aimed at understanding the relationship of ticks to climate, and ignore other basic aspects, such as vegetation patterns or host abundance, that are also involved in delineation of the “ecological” preferences of a tick species or population. Species occurrence can be predicted by inclusion of appropriate climate variables in what are commonly referred to as climate suitability models (CSMs): the relationships are generalized from a sample of correlations of species presence with specific values of environmental variables. While it is well recognized that the climate niche space occupied by a species across its geographic range may vary, this is rarely considered in current modeling approaches despite its obvious importance. When regional climate niche variations occur, a CSM derived for a particular area may not apply to other areas, and a model derived from a large area may have comparatively weak local predictive power. A widely held assumption in traditional models of tick distribution is that responses of species to environmental gradients are unimodal and symmetrical. Thus, climate suitability is predicted to decline from central (and ecologically optimal) areas of a species' range towards the periphery. In suboptimal conditions, a species may compensate for physiological stress by a shift in niche position. For wide-ranging species, suitable ecological conditions may vary considerably between different regions within the range.

However, these CSM are unsuitable if an adequate understanding of the factors operating over the transmission of a disease is necessary. The many variables involved in such processes, like hosts, densities of questing infected ticks, and a perception of the small scale of foci, are only adequately addressed with models designed to describe seasonal dynamics. While some models with biological content have been produced for tick species such as *Boophilus microplus* [44], *Amblyomma americanum* [45], and *Ixodes scapularis* [46], none are currently available for European ticks. Such models are a priority to adequately understand the impact of climate change on tick populations, provided that adequate data are used for important components such as host densities and microhabitat suitability. However, despite the absence of such data in climate suitability models, these models have proved useful in the elucidation of tick-borne disease foci for the recent outbreak of CCHF in Turkey [47].

### 4.2. Recent (100 Years) Changes in Climate Suitability for Ixodes ricinus

**Figure 3:**
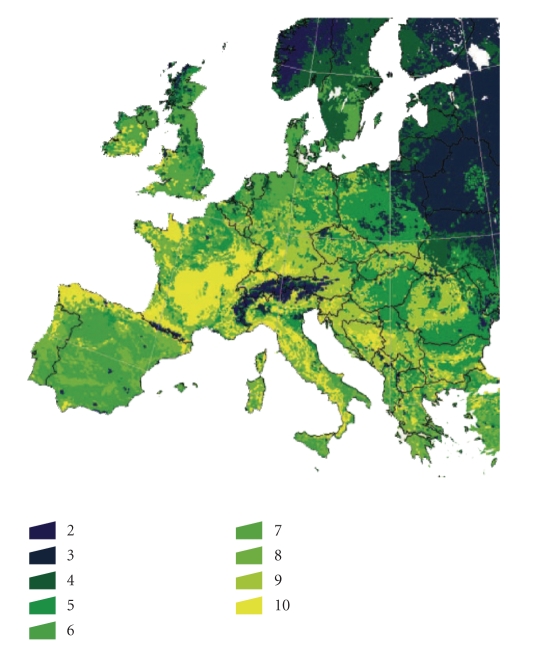
The vegetation-derived clusters as recognized over the western Palaearctic. The image was obtained from a yearly series of monthly satellite images, capturing the Normalized Difference Vegetation Index (NDVI): a measure of the photosynthetic activity of the vegetation. These images were subjected to a cluster analysis according to the monthly NDVI features to obtain 10 categories (category 1 is water and not displayed in the picture). (Estrada-Peña et al. 2006 [48], with permission from *Medical and Veterinary Entomology*).

Both regional climatic requirement variations and the existence of “demes” (defined as populations of closely related interbreeding organisms of the same species with differing responses to the wide array of climate factors occurring across the geographical range of the species) have been demonstrated for *I. ricinus* [47] (Figure 3).

The study revealed at least 10 distinct *I. ricinus* groups with a pattern of distribution closely overlapping with the presence of previously reported phenotypic forms of the species, and is in general agreement with a 16S mitochondrial rDNA study of genetic variation in *I. ricinus* [49]. The importance of these findings is that the climate and vegetation features correlated with the genetic groups in the whole tick metapopulation, with different populations having specific climatic requirements and unique genetic fingerprints. A study on the changes in climate suitability for *I. ricinus* in the western Palearctic has been carried out using deme-derived models based on the different populations recognized in the Palaearctic [50]. The distribution records available for the different demes were used to build partial models (i.e., applied to regions of the whole distribution range) from which a complete map for the whole region was produced. The study used a long (1900–1999) series of climate data at coarse resolution (10 minutes of arc) to examine the trends in climate and to estimate sustained variations in climate suitability for *I. ricinus*. While some areas showed a deterministic (i.e., continuous) tendency towards increasing or decreasing suitability for the tick, others showed unambiguous cycles of climate suitability, termed areas of random walk. In these, populations of the tick may undergo periodical variations in their geographical range as a consequence of cyclic changes in climate.

**Figure 4:**
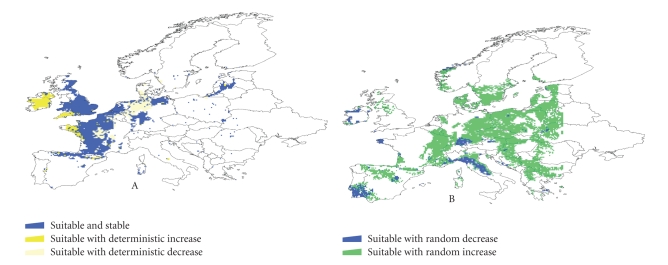
An analysis of the long-term changes in climate suitability for the tick *Ixodes ricinus* in Europe (1900–1999). A temporally extensively gridded dataset was subjected to a temporal analysis to understand how climate has changed in 100 years and how this trend affected the climate suitability for the tick. Areas are divided into suitable and unsuitable (the last, without colors in the figure). In the panel “A,” the area marked as suitable and stable means no changes in suitability for the tick. Deterministic increase or decrease means a continued trend towards increasing or decreasing climate suitability. Panel B shows the areas where random walk trend has been observed. These areas are subjected to periodic cycles of climate, thus allowing cycles of increasing or decreasing climate suitability for the tick. (Estrada-Peña and Venzal 2006 [50], with permission from *Ecohealth*).

This analysis suggests that while climate suitability for *I. ricinus* did not change in a large area of Europe during the 100-year study period, it increased in specific geographically limited locations and decreased in others (Figure 4).

These changes are not recent and are associated with yearly and summer rainfall patterns rather than with temperature. The reported increased abundance of *I. ricinus* in parts of Europe (e.g., Sweden [11]) coincides geographically with the regions where a recent increase in climate suitability has been detected, within zones having a marked random walk tendency. Thus, the observation of higher tick abundance in recent years may not be due to a permanent shift in tick populations, but rather because the long-term climate cycle, which varies on a wide timescale, has been in a phase that is favorable to tick survival. No single variable was consistently associated in the study period with changes in climate suitability across sites where random walk was detected. The absence of a single regulatory variable seems to be connected with the different climate niche experienced by the tick populations in their distribution area. Thus, rainfall and temperature have different regulatory abilities according to the portion of the tick's climate envelope represented in a given area.

### 4.3. An Overview of the Climate Suitability for Ticks in the Mediterranean Region

The Mediterranean region is expected to experience profound changes in climate [51], and furthermore, its close proximity to the African continent makes it a particularly sensitive area to invasion by ticks currently restricted to northern Africa. It is, therefore, of interest to explore the predicted impact of climate changes on the suitability for ticks. The ticks involved (genera *Boophilus*, *Dermacentor*, *Rhipicephalus,* and *Hyalomma*) find their maximum suitability in Mediterranean type vegetation, including areas with cold winters and dry summers, and are prevalent in wide areas of that region, extending well into the Middle East. Most of them are vectors of pathogens of animals and humans. Predicted changes in climate are also expected to have a serious impact on the vegetation structure, driving the area toward a more open, brush-like vegetation, where these ticks find their ecological optima.

**Figure 5:**
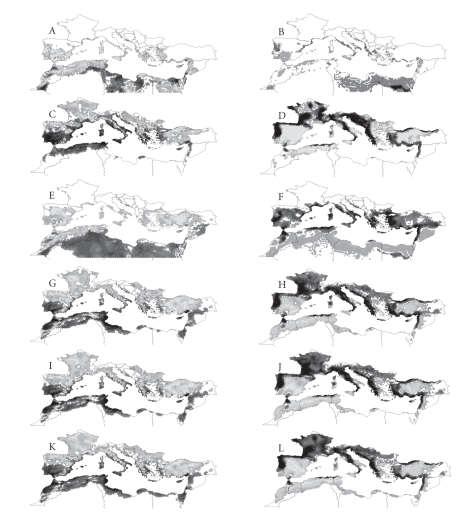
Predicted geographic impact (habitat suitability turnover) of different climate change scenarios. The maps shows the forecasted changes in habitat suitability for different tick species, with changes in temperature (left column) or rainfall (right column) analyzed by consensus analysis (a statistical method of classification using multiple input variables) to show the most coherent response to a range of changes in predictor variables. Dark shades of grey indicate increased climate suitability following a decrease in the predictor variable scenario (temperature or rainfall). Light shades of grey indicate increased climate suitability following an increase in the predictor variable. A and B: *B. annulatus*; C and D: *D. marginatus*; E and F: *H. excavatum*; G and H: *H. marginatum*; I and J: *R. bursa*; K and L: *R. turanicus*. (Estrada-Peña and Venzal 2007 [52], with permission from *Journal of Medical Entomology*).

An estimation of the climate niches for each of these ticks has already been published [52]. Global climate models remain relatively coarse in terms of spatial resolution; this compromised the desired resolution of that analysis. To address this problem, new climate layers were created with monthly increases and decreases in temperature of 1 and 2°C, and monthly variations in rainfall of 60, 80, 120, and 140% of actual values. For each combination of temperature and rainfall, the climate suitability for each species in the region was evaluated. The baseline climate suitability was used as a framework to compute the impact of the different changes of climate over the expansion or retraction of the geographical range for each species. The expected impact is displayed in Figure 5.

For *B. annulatus* and *H. excavatum*, an increase in temperature would lead to an increase in suitability within regions of northern Africa and limited parts of southern Europe. Changes in rainfall are predicted to have little geographic impact on *B. annulatus* (which in any case is a one-host-tick that is much less exposed to ambient conditions than three-host-ticks), but they show a clear effect on *H. excavatum.*

In the case of *D. marginatus*, a decrease in temperature results in an increase in the extent of adequate suitability in northern Africa, most of coastal southern Europe, and wide areas of southern Spain, whereas an increase in temperature would result in a northward expansion of the suitable habitat for this species. Changes in rainfall result in similar effects to those described for the above-mentioned changes in temperature. Large areas of Europe would potentially be affected by an increase in the climate suitability for *Rhipicephalus* spp. and *H. marginatum* after an increase in temperature and decrease in rainfall. Decreasing temperatures in Europe are predicted to result in habitat loss for *B. annulatus, R. bursa,* and *H. marginatum*. Again, we must stress that this is an evaluation of the climate suitability for these tick species, since changes in vegetation, host availability, and animal movements were not included in the models.

## 5. Effects on Tick-Borne Diseases in Europe

All the tick species considered in the earlier sections are important vectors of disease and an increasing incidence of these diseases is the most significant potential outcome of climate changes that affect ticks, directly or indirectly. As described above, there is little doubt that *Dermacentor reticulatus* is currently extending its range and this is reflected in the occurrence of canine babesiosis (caused by *Babesia canis canis*) in new areas (24, 25, 26, 27, 28). *Hyalomma marginatum* is one of the vectors of Crimean-Congo hemorrhagic fever (CCHF), which occurs in parts of Africa, Asia, the Middle East, and south-east Europe. The largest epidemic on-record occurred in Turkey, which started in 2002 and is still ongoing [47], and further north, a recent outbreak was reported in the Balkans, another known endemic area [53], but there is no evidence as yet that this disease is spreading further northwards. However, as discussed above, the potential for the introduction and establishment of vector populations in areas of predicted climate suitability is increasing.

*Rhipicephalus sanguineus* is the primary vector of Mediterranean-spotted fever (a rickettsial zoonosis caused by *Rickettsia conorii*), and also *Ehrlichia canis* and *Babesia canis vogeli* (causing, resp., rickettsial and protozoal diseases of dogs). Although *R. sanguineus* can establish in kennels in central and northern European latitudes, thus potentially causing short-lived localized disease outbreaks, sufficient survival in the external environment does not seem to occur for significant disease transmission in northern temperate Europe. Nevertheless, concerns about its possible introduction and establishment have prompted the authorities in some countries, such as the United Kingdom and Ireland, to make treatment against ticks a component of the pet-passport scheme.

*Ixodes ricinus* is the most abundant and widespread tick in Europe and together with the Eurasian species, *I. persulcatus*, transmits Lyme borreliosis (LB) and tick-borne encephalitis (TBE). LB occurs at a relatively high incidence for a zoonotic disease, ranging from 155 per 100,000 in Slovenia to 0.6 per 100,000 in Ireland [54]. However, it is difficult to statistically relate LB incidence to climate change because the reliability of incidence data is uncertain due to diagnostic problems and limited or absent reporting in most countries. Nevertheless, in some regions, it has proved possible to relate disease incidence to climate, and a positive association of incidence with mild winters and warm, humid summers was reported in southern Sweden [55]. The suggested effect of mild winters is possibly due to an extension of the tick activity season, resulting in increased numbers of infected ticks becoming available in the following year. Warm humid summers might result in a more efficient transmission by the vector, but there is limited evidence for this and the authors suggest that the observed increased LB incidence may be due to increased human exposure in these conditions.

Since TBE is notifiable in most countries where it occurs, current incidence data are more reliable than those for LB, even though reporting standards differ between countries and there are increasing differences in TBE vaccination rates between countries. Increases in Swedish cases since the mid-80s were associated with two consecutive years with milder winters, earlier arrival of spring and prolonged autumn periods with temperatures above 5–8°C [13]. The possibility that this is caused by climate effects on ticks is suggested by the northward extension of *I. ricinus* distribution [11, 12]. Similarly, an upward movement of the TBE prevalence altitude ceiling correlating with increasing temperatures has been reported [15, 56], which accords with reports of increasing numbers of active ticks at higher altitudes [15].

Detecting climatic effects on the incidence of LB and TBE in areas close to the latitude and altitude distribution limits of the vector is less complex than in other parts of Europe where the direct and indirect impacts of climate change on disease incidence are often confounded by other factors. Such studies should either be based on reliable long-term historical datasets in an area (e.g., the 40 year surveillance program for TBE in Stockholm County, Sweden [13]) or based on data from different regions or countries, collected with similar methodologies (e.g., vector sampling and analysis) and subject to similar variations (e.g., TBE vaccination rates and reporting criteria). There is a need for a pan-European surveillance network, for example based on the abundance of infected vectors, as suggested by WHO [57].

Randolph et al. [58] correlated the occurrence of synchronous activity of *I. ricinus* larvae and nymphs (resulting in cofeeding transmission) with that of TBE cases, and also reported a relationship between these two variables and the rate of decline of autumn temperatures. It, therefore, seems likely that climate change will affect the dynamics of TBE transmission, thus altering the distribution of the disease [59], but the exact mechanisms of this interaction remain to be elucidated.

Despite the ready availability of data on TBE incidence, a firm causal relationship with climate change remains elusive. A detailed study of the records of TBE incidence over the last 2-3 decades in Estonia, Latvia, and Lithuania showed that although TBE incidence rose dramatically in some areas, there was so much heterogeneity in the data that it was impossible to identify climate change as the main factor driving increased disease incidence. It was concluded that the many socioeconomic changes arising from the end of Soviet rule probably acted synergistically with climate factors to increase TBE incidence [60].

## 6. Conclusions

Transmission of infection occurs when there is an overlap of activities between reservoir, vector, and humans, and differs according to the pathogens and the location. Climate change may impact all of these stages and their interactions. Although changes in climate and in the length of the different seasons will directly affect tick survival, activity, and development, there is no good evidence that rising temperatures will result in a greater abundance of ticks by simply increasing rates of development; rather changes in development rates will make tick cohorts available to different diapause windows (largely determined by day length), thus changing patterns of seasonal activity [38]. Indirect effects of climate change will impact the number of infected ticks by affecting vegetation. For example, a warming climate in central Europe is likely to result in a decrease of Norway spruce (*Picea abies*) and the areas involved will probably be colonized by beech (*Fagus sylvatica*) [61], the fallen leaves of which provide a favorable microclimate for survival of the free-living tick stages. Additionally, climate change will also have indirect effects on tick-borne pathogen transmission by affecting the survival and abundance of tick maintenance hosts, such as deer, and pathogen-reservoir hosts such as rodents and birds. Climate change may also influence disease risk by affecting the long-term use of land (e.g., farming, tourism, etc.), and weather patterns have an effect by influencing short-term human behavior such as picnics and mushroom picking. Climate effects are more easily noticeable close to the geographical distribution limits of both vector and disease. The magnitude of the effects of climate change in an endemic area depends on local conditions and vulnerability, and is determined not only by ecological conditions but may be influenced by socioeconomic factors, human migration and settlement, ecosystems and biodiversity, migrating patterns of birds, land-use and land cover changes, human cultural and behavioral patterns, and immunity in the population. Since some of these conditions are in turn influenced by climate change, a complex chain of processes exists that makes the precise factors responsible for changes in disease incidence often difficult to determine [54]. A further difficulty in determining future scenarios is presented by the fact that the predominant tick species in Europe, *Ixodes ricinus* is extremely flexible and adaptable and can exhibit rather different seasonal activity even in adjacent parts of its geographical range. Much current research effort attempts to match datasets collected for different purposes and in order to reduce confounding variables, it is evident that data from long-term studies on disease incidence, tick biology, tick distribution and tick abundance, host abundance and distribution, and relevant vegetation biology, specifically in relation to climate change, are required [62]. Such data will permit the development of models to predict future tick-borne disease scenarios, which take account of dynamic biological processes instead of simply the likelihood of occurrence of climate suitability for particular tick species.
